# CD24 Expression Dampens the Basal Antiviral State in Human Neuroblastoma Cells and Enhances Permissivity to Zika Virus Infection

**DOI:** 10.3390/v14081735

**Published:** 2022-08-06

**Authors:** Kritika Kedarinath, Candace R. Fox, Erin Crowgey, Joseph Mazar, Peter Phelan, Tamarah J. Westmoreland, Kenneth A. Alexander, Griffith D. Parks

**Affiliations:** 1 Burnett School of Biomedical Sciences, College of Medicine, University of Central Florida, Orlando, FL 32827, USA; 2 College of Medicine, University of Central Florida, Orlando, FL 32827, USA; 3 Research Department, Nemours Children’s Hospital Delaware, Wilmington, DE 19803, USA; 4 Department of Biomedical Research, Nemours Children’s Hospital, Orlando, FL 32827, USA

**Keywords:** CD24, zika virus, type I interferon, antiviral responses, permissivity

## Abstract

Zika virus (ZIKV) exhibits distinct selectivity for infection of various cells and tissues, but how host cellular factors modulate varying permissivity remains largely unknown. Previous studies showed that the neuroblastoma cell line SK-N-AS (expressing low levels of cellular protein CD24) was highly restricted for ZIKV infection, and that this restriction was relieved by ectopic expression of CD24. We tested the hypothesis that CD24 expression allowed ZIKV replication by suppression of the antiviral response. SK-N-AS cells expressing an empty vector (termed CD24-low cells) showed elevated basal levels of phosphorylated STAT1, IRF-1, IKKE, and NFκB. In response to exogenously added type I interferon (IFN-I), CD24-low cells had higher-level induction of antiviral genes and activity against two IFN-I-sensitive viruses (VSV and PIV5-P/V) compared to SK-N-AS cells with ectopic CD24 expression (termed CD24-high cells). Media-transfer experiments showed that the inherent antiviral state of CD24-low cells was not dependent on a secreted factor such as IFN-I. Transcriptomics analysis revealed that CD24 expression decreased expression of genes involved in intracellular antiviral pathways, including IFN-I, NFκB, and Ras. Our findings that CD24 expression in neuroblastoma cells represses intracellular antiviral pathways support the proposal that CD24 may represent a novel biomarker in cancer cells for susceptibility to oncolytic viruses.

## 1. Introduction

There is intense interest in understanding the role of cellular factors that modulate the permissivity of a cell to a viral infection, with a particular interest in RNA viruses that have the potential to rapidly evolve to fit new cellular environments. Several factors can influence viral selectivity for a cell or tissue [1], including: (a) the presence of required specific host cell entry receptors (e.g., CD4, CCR5, and CXCR4 for HIV-1), (b) the availability of host factors required for intracellular replication (e.g., nuclear factors for hepatitis B virus), and (c) the baseline and induced antiviral state within the infected cell.

The type I interferon (IFN-I) pathway is an important mediator of innate immunity, which can play an impactful role in regulating permissivity for many viruses [1,2]. The canonical IFN-I pathway consists of two main phases—production and signaling—resulting in increased expression of a wide range of interferon-stimulated genes (ISGs) whose products can inhibit the viral growth cycle at various stages [2]. The IFN-I production phase is characterized by the detection of viral components, such as dsRNA, by host pattern-recognition receptors, such as melanoma differentiation-associated protein 5 (MDA-5) and retinoic acid-inducible gene I (RIG-I). This triggers activation of Interferon Regulatory Factor (IRF) proteins, which ultimately results in the transcription of IFN-I genes leading to their production and secretion from the infected cell. Some IRFs, such as IRF-1, can directly activate ISG expression to induce an antiviral state [3]. Secreted IFN-I can bind in an autocrine or paracrine manner to interferon receptors (IFNAR) on host cells to trigger IFN-I signaling. A critical step in the signaling phase involves the phosphorylation and heterodimerization of the Signal Transducer and Activator of Transcription 1 and 2 (STAT1 and STAT2) proteins. Translocation of the phosphorylated STAT complex to the nucleus ultimately leads to the transcription of ISGs capable of inducing an antiviral state within the infected cell and uninfected neighboring cells [2].

ZIKV has emerged in recent years as a public health concern after triggering a global health emergency in 2016 [4]. A member of the *Flaviviridae* family, the virus consists of a positive-sense RNA genome that codes for three structural proteins: capsid (C), membrane (M), and envelope (Env), along with seven nonstructural (Ns) proteins [5]. Various human and animal studies have revealed that ZIKV has a broad selectivity for infections of specific cells and tissues, including retinal, neural, reproductive, and fetal tissue [6]. ZIKV infections can also be modulated by IFN-I responses [6].

In our previously published work, we found that many neuroblastoma cell lines were permissive to ZIKV infection in vitro [7]. SK-N-AS cells were a striking exception to this permissivity, as these cells were highly restricted for ZIKV infection. Transcriptomics analysis revealed that host cellular protein CD24 was present at moderate to high levels in the ZIKV-permissive neuroblastoma cell lines (e.g., IMR-32, CHLA-42, SMS-KAN). By contrast, CD24 was minimally expressed in the poorly permissive SK-N-AS cells. Most importantly, ectopic expression of CD24 in the ZIKV-restricted SK-N-AS cells converted them into a cell line that was now permissive to ZIKV infection. Thus, ectopic CD24 expression alone was sufficient to increase the permissivity of an otherwise restricted ZIKV infection [7]. The host cell protein CD24 is expressed in a variety of progenitor and developing hematopoietic (e.g., B and T cells, neutrophils, dendritic cells, macrophages) and non-hematopoietic (e.g., neural cells, keratinocytes, epithelial cells) cell types [8]. CD24 is a ~15 kDa sized polypeptide that is heavily glycosylated, typically anchored to membranes via a glycosyl phosphatidylinositol (GPI) link. Expression of CD24 impacts a wide range of cellular functions, including neural development, cellular transcription, activation of adaptive immunity, inflammation, apoptosis, and cell proliferation [8,9]. Here, we tested the hypothesis that host cellular protein CD24 can modulate the antiviral state of human neuroblastoma cells in vitro and can determine the permissivity of these cells to infection with ZIKV and other distinct classes of RNA viruses.

## 2. Materials and Methods

### 2.1. Cell Lines, Viruses, and Infections

Cultures of SK-N-AS, Vero, BHK-21, and A549 cells were grown and maintained in Dulbecco’s modified Eagle medium (DMEM) supplemented with 10% heat-inactivated fetal calf serum (HI FBS, Gibco, Thermo Fisher Scientific, Waltham, MA, USA) at 37 °C and under humidified 5% CO_2_ atmosphere conditions. CD24-low and CD24-V1 cells were generated as described previously [7]. To derive a homogenous CD24-high cell line, single-cell sorting was performed by suspending 10^7^ cells/mL CD24-V1 cells in a mixture of phosphate-buffered saline (PBS) and 5 µL of Alexa Fluor^®^ 647 anti-human CD24 antibody per 10^7^ cells/mL (Biolegend, San Diego, CA, USA). Cells were incubated for 15 min (min) in the dark and washed and resuspended in DMEM supplemented with 2% HI FBS and 1 mM EDTA. Cells were then strained into polystyrene tubes, and single-cell sorting was performed using BD FACSAria™ IIu (BD Biosciences, San Jose, CA, USA). After sorting, cells were collected and grown in DMEM supplemented with 30% HI FBS and Penicillin–Streptomycin, before being maintained under the conditions described above. SK-N-AS cell lines expressing variants of CD24 (CD24-low and -high) were cultured with 6 µg/mL blasticidin (Invivogen, San Diego, CA, USA). A549 cells expressing a nuclear red fluorescent protein (A549-NLR cells) were purchased from Incucyte (Sartorius, Bohemia, NY, USA) and maintained with selection at 1 µg/mL Puromycin (Invivogen).

The PIV5 mutant PIV5-P/V-CPI− (PIV5-P/V mutant) expressing GFP was generated and grown in Vero cells as previously described [10]. The MR766 strain of Zika virus (ZIKV) (ATCC–VR84, Manassas, VA, USA) was propagated in Vero cells using a low multiplicity of infection (MOI 0.1). The Orsay Strain of VSV expressing EGFP was the kind gift of Doug Lyles (Wake Forest University, Winston-Salem, NC, USA) and was grown in BHK-21 cells. For infections, plates of cells were either infected with the virus for 1 h (hour) in DMEM containing 10% bovine serum albumin (BSA) or mock-infected with the indicated medium only. After the incubation, cells were washed with PBS and incubated in DMEM supplemented with 2% HI FBS.

### 2.2. Plaque Assays

ZIKV viral titers were determined on Vero cells using plaque assays. Briefly, 6-well plates of Vero cells were infected with serial dilutions of virus in DMEM supplemented with 2% HI FBS. After 1 h of incubation, cells were washed with PBS and overlayed with a 1:1 solution mixture of 0.6% agarose and DMEM supplemented with 2% HI FBS. After the overlay had solidified, plates were incubated at 37 °C in a humidified 5% CO_2_ incubator for six days (d). The agarose overlay was removed, and cells were fixed and stained for 30 min (minute) with a 1% crystal violet solution containing 3.7% formaldehyde, 20% ethanol, and PBS.

### 2.3. Immunostaining and Fluorescence Microscopy

Cells were seeded in 48-well plates and treated as described in figure legends. Cells were fixed and permeabilized with 2% paraformaldehyde and 100% ice-cold methanol. Cells were blocked in buffer containing PBS, 5% HI FBS, and 0.3% Triton™X-100 and then incubated with antibodies (as indicated in the figure legends) in antibody dilution buffer containing PBS, 1% BSA, and 0.3% Triton™X-100, followed by secondary staining with Alexa Fluor^®^ 568 (Thermofisher Scientific, Waltham, MA, USA). Antibodies against STAT1 and IRF-1 were obtained from Cell Signaling Technology (Danvers, MA, USA) and utilized at dilutions according to the manufacturer’s recommendations. To visualize and stain for nuclei, 4; 6-Diamidino-2-pherylindole (DAPI) stain was included. To visualize levels of CD24, cells were fixed with 2% paraformaldehyde diluted in 3% BSA and PBS. For intracellular staining, cells were additionally permeabilized with a solution containing 10% saponin, PBS, and BSA. Cells were incubated with a CD24 monoclonal antibody (SN3) (Invitrogen, Thermofisher Scientific), followed by secondary staining using α-mouse Alexa Fluor^®^ 568 (Thermofisher). Fluorescence images were captured on a 20× objective lens using the Keyence BZ-X800 (Keyence, Itasca, IL, USA) according to optimal exposure times for sample conditions. Images were then analyzed on the Keyence BZ-X800 analysis software. (Keyence, Itasca, IL, USA).

### 2.4. Quantification of Virus Infection, Cell Viability, and CD24

Cells were cultured in 24-well plates and treated as described in the figure legends. To quantify non-viable cells, medium was collected, and adherent cells were trypsinized, centrifuged, and washed with PBS before staining with propidium iodide (PI, BD Bioscience, Franklin Lakes, NJ, USA). To determine the percentage of cells that express CD24 extracellularly, cells were stained with 4 µg/mL of Alexa Fluor^®^ 647-conjugated anti-human CD24 (Biolegend), or with corresponding isotype control Alexa Fluor^®^ 647 mouse IgG2a-κ isotype control antibody (Biolegend). To quantify ZIKV infection, cells were fixed and permeabilized using eBioscience Intracellular Fixation and Permeabilization Buffer, according to the manufacturer’s instructions (Invitrogen, Thermofisher Scientific). Staining was then performed using Flavivirus group antigen–antibody (D1-4G2-4-15; Novus Biologicals, Littleton, CO, USA) and Alexa Fluor^®^ 488 (Thermofisher Scientific) to determine ZIKV Envelope protein expression. Cells were analyzed using flow cytometry with the CytoFLEX system (Beckman Coulter, Brea, CA, USA) by recording 10,000 independent events, and subsequent analyses were performed using CytExpert Version 2.4.0.28 (Beckman Coulter, Brea, CA, USA) software.

### 2.5. Exogenous Treatment with IFN-I or IFN-III and Quantification of IFN-I Levels

Media were removed from cells before treatment with Universal IFN-I or human IL-29/IFN Lambda 1 (IFN-III) (both from PBL Assay Science, Piscataway, NJ, USA) diluted in DMEM supplemented with 2% HI FBS and at the concentrations indicated in the figure legends. Prior to quantifying biological levels of IFN-I from the supernatant of infected cells, inactivation of virus was performed. Briefly, the indicated supernatant was acid treated with 1N HCl at room temperature for 30 min, followed by titration with 1N NaOH to achieve neutralization of acid. IFN-I was subsequently quantified in virus-inactivated supernatant using HEK-Blue™ IFN-α/β indicator cells (Invivogen) and QUANTI-Blue™ reagent (Invivogen), as previously described [11].

### 2.6. Reverse Transcription and Real-Time PCR

Six-well dishes were treated as indicated in the figure legends, and RNA extraction was performed using TRIzol (Invitrogen) as described previously [12]. TaqMan^®^ Reverse Transcription Reagents (Applied Biosystems, Foster City, CA, USA) were used to obtain cDNA from 1 µg of total RNA (as per the manufacturer’s instructions). Bio-Rad CFX Connect Real-Time and Fast SYBR^®^ FAST Green Master Mix (Applied Biosystems) were used to perform quantitative real-time PCR. The primer sequences utilized are shown in Table 1, below:

Forward and reverse primer sequences for genes *IFIT1*, *IFITM1*, and *MxA* were obtained from previously published studies [13,14,15]. For determining the DNA Copy Number for the ZIKV *NS1* gene, plasmid pcDNA3.1 encoding *NS1* linked to FLAG sequence was generated (details available on request). Relative gene expression and total expression values were generated using CFX Manager™ 3.1 Software (Bio-Rad, Hercules, CA, USA).

### 2.7. Nuclear Extraction

Cells were grown in dishes (60 mm or 100 mm) and treated as indicated in the figure legends. Nuclear and cytoplasmic extracts were then obtained using the Active Motif Nuclear Extract Kit (Active Motif, Carlsbad, CA, USA) according to the manufacturer’s instructions. Protein quantification of the extracts was performed using Pierce™ BCA Protein Assay Kit (Thermofisher Scientific). An amount of 4 µg (from 60 mm dish) or 10 µg (from 100 mm dish) of protein was lysed in 1X protein lysis buffer (Cell Signaling Technology, Danvers, MA, USA) and analyzed via Western blotting.

### 2.8. Western Blotting and Densitometry

Cells treated as described in the figure legends were lysed using 1X protein lysis buffer (Cell Signaling Technology, Danvers, MA, USA), resolved on 8–12% sodium dodecyl sulfate-polyacrylamide gel electrophoresis (SDS-PAGE) gels and transferred to nitrocellulose membranes. Samples were then probed with antibodies as indicated in the figure legends or with anti-β-actin antibody (Sigma-Aldrich, St. Louis, MO, USA). Antibodies against pSTAT1, STAT1, PARP, α-tubulin, IRF-3, IRF-1, NFκB1, NFκB2, and IKKε were obtained from Cell Signaling Technology and utilized at dilutions recommended by the manufacturer. Blots were then visualized by horseradish-peroxidase-conjugated antibodies and SuperSignal™ West Pico PLUS chemiluminescent substrate (ThermoScientific, Rockford, IL, USA). Densitometry analysis on blots were performed using Image Studio™ (Version 4, LI-COR Biotechnology, Lincoln, NE, USA) as per the manufacturer’s instructions.

### 2.9. Supernatant Transfer

CD24-low and -high cells were seeded in 24-well dishes and cultured for 3 d. Culture medium was then collected and diluted with DMEM supplemented with 10% HI FBS, as described in each figure legend. Freshly cultured naïve CD24-high cells were then individually treated with diluted culture medium for ~16 h. As controls, CD24-high cells were either left untreated or treated with Universal IFN-I.

### 2.10. Global Transcriptomics

Cells were seeded in 6-well plates and treated as indicated in the figure legends. At 20 and 40 h post-infection (hpi), cellular and viral RNA was extracted using the RNeasy Plus Mini Kit (QIAGEN, Germantown, MD, USA) as per the manufacturer’s instructions, and subsequent quality control was performed using Nanodrop (Thermo Scientific) to ensure that an A260/280 ratio of ~2.0. RNA samples were then diluted to 50 ng/mL and shipped to Genewiz^®^ (South Plainfield, NJ, USA) for RNA-Sequencing. To prepare sequencing libraries with the Illumina RNA prep using polyadenylated RNA selection, 1.5 µg of RNA was utilized. RNA integrity, library size, and concentrations were evaluated on the Agilent TapeStation and with the Qubit instrument (LifeTech, Thermofisher Scientific, Waltham, MA, USA) according to GeneWiz^®^ specifications. Libraries were then sequenced on an Illumina HiSeq 2500 2 × 150 bp with a single index, and paired-end reads were generated.

Raw FASTQ files were processed using the “new Tuxedo” pipeline [16]. Briefly, reads that contained adapter sequences and low-quality reads were removed using Trimmomatic. Subsequent clean reads were further analyzed using FastQC to ensure only high-quality reads were processed. Clean reads were then aligned to the human genome (version GRCH38) using HISAT2. Stringtie and Ballgown in R were then utilized to identify differentially expressed genes, and statistical analyses were performed. Files are available via NCBI accession PRJNA858025: Zika Infection in SKNAS VO and CD24 Cells Sequencing.

Fold changes of genes involved in the IFN-I induction and signaling pathways were visually represented via heat maps generated in GraphPad^®^ Prism software—GraphPad Prism Version 9.3.1.471 (GraphPad Software, San Diego, CA, USA) with * indicating a *p*-value less than 0.05. For the ZIKV infection longitudinal studies, genes that were only statistically significant at one time point were identified. Pathway enrichment analysis was performed comparing mock-infected CD24-high to CD24-low cells by inputting statistically significant genes with fold changes greater than 2.5 or less than 0.25 into the reactomeFI App in Cytoscape [16,17].

### 2.11. Figures, Statistics, and Images

Mean values and standard deviations were generated using GraphPad^®^ Prism software. *p* values were generated using 2-way ANOVA. In all figures, *, **, and *** indicate *p*-values < 0.033, <0.002, and <0.001, respectively (*p*-value style was set to APA). Graphs were generated using GraphPad^®^ Prism software. Experimental setup was created with BioRender.com.

## 3. Results

### 3.1. CD24 Expression Increases ZIKV Spread and Infectivity in a Neuroblastoma Cell Line

**Figure 1:**
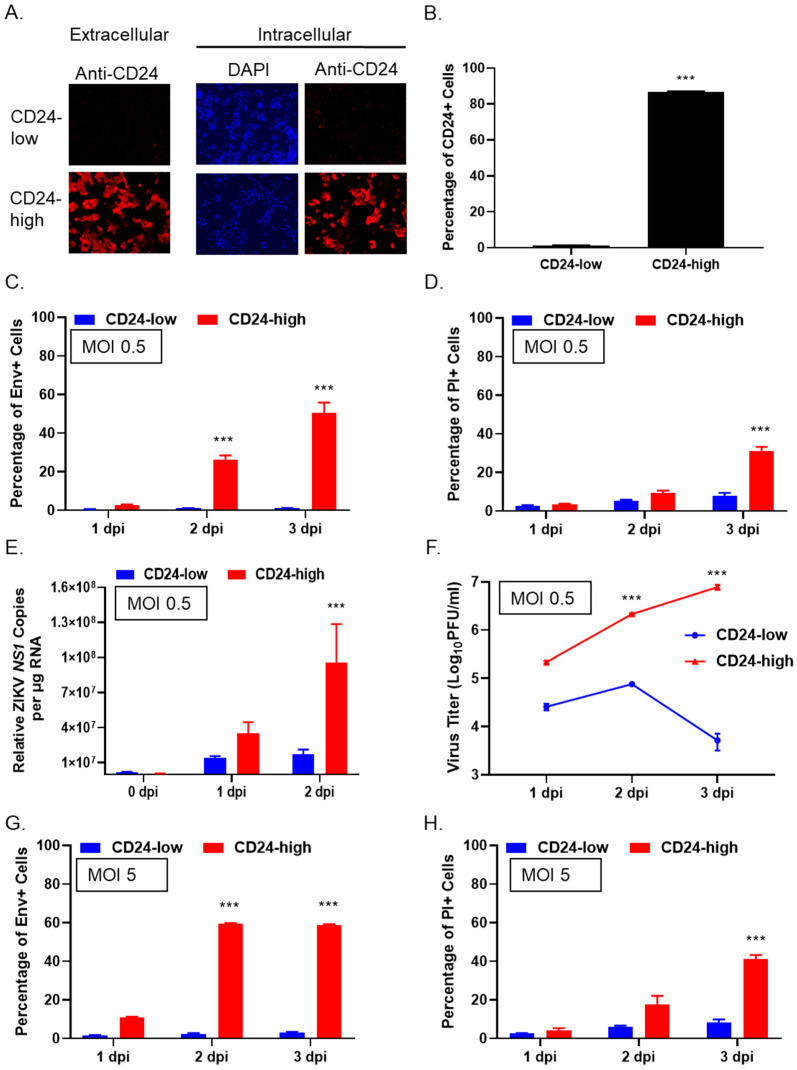
Ectopic expression of CD24 in SK-N-AS cells enhances low MOI spread of ZIKV infection. (**A**,**B**) CD24-low and -high cells were analyzed by 4; 6-Diamidino-2-pherylindole (DAPI) staining (blue) or immunostaining with anti-CD24 antibody (red) (**A**) or by flow cytometry (**B**) for CD24 expression. Data are a representation of two separate experiments. (**C**–**F**) CD24-low and -high cells were infected with ZIKV at a low MOI of 0.5. Flow cytometry was performed at indicated time points to determine the percentage of infected (Env+) cells (**C**) or non-viable (PI+) cells (**D**). Total cellular RNA was isolated from infected CD24-low and -high cells at indicated time points, and gene levels of *NS1* were determined via qRT-PCR. Data are expressed as Relative ZIKV *NS1* copies per µg of analyzed RNA (**E**). Media were harvested at indicated time points from the infected cell cultures and analyzed by plaque assays to determine levels of infectious virus. Viral titers were expressed as PFU/mL on a logarithmic scale and normalized to 10^6^ infected cells (**F**–**H**). CD24-low and -high cells were infected at a high MOI of 5, and the percentage of Env+ (**G**) and PI+ (**H**) cells were determined at the indicated days post-infection (dpi). All figures’ values are the mean of three samples, with error bars representing standard deviation. *** indicates *p*-value < 0.001.

SK-N-AS is a non-MYCN amplified metastatic neuroblastoma cell line that endogenously expresses minimal levels of CD24 [7,18]. In our prior work [7], SK-N-AS cells were transfected to stably harbor an empty vector as a control (CD24-low cells) or a vector that expresses elevated levels of CD24 (CD24-high cells). Flow cytometry and cell sorting were used to enrich the CD24-high cell population for homogeneous and high-level CD24 expression. To evaluate the distribution of CD24 within the cell populations, immunofluorescence was performed by extra- and intracellular staining of CD24-low and -high cells with a monoclonal anti-CD24 antibody (Ab). As shown in Figure 1A, CD24-low cells showed minimal to undetectable staining with anti-CD24 Ab, while CD24-high cells showed a high level of both extra- and intracellular staining for CD24. Quantification of extracellular CD24 expression by flow cytometry showed that CD24-low cells had undetectable levels of CD24, while ~80% of the CD24-high cells exhibited CD24 expression (Figure 1B).

To determine how levels of CD24 expression affected ZIKV infection, CD24-low and -high cells were infected at a multiplicity of infection (MOI) of 0.5, and the percentage of cells expressing ZIKV envelope protein (Env+ cells) was quantified by flow cytometry on 1, 2, and 3 days post-infection (dpi). Additionally, cell viability was monitored by quantifying positive cells for propidium iodide staining (PI+ cells). Infection of CD24-low cells showed a minimal percentage of Env+ cells (~1–5%) across the time course (Figure 1C), compared to infection of CD24-high cells, which led to ~50% Env+ cells by 3 dpi. Correspondingly, <10% of infected CD24-low cells appeared to be PI+ across the time course, while the percentage of CD24-high cells that were PI+ increased to ~30% by 3 dpi (Figure 1D).

Viral RNA load in infected cell populations was quantified by use of RT-PCR (qRT-PCR) to measure ZIKV *NS1* gene expression. As shown in Figure 1E, infected CD24-low cells expressed low levels of *NS1* across the entire time course. In contrast, CD24-high cells exhibited a time-dependent increase in *NS1* gene expression, peaking at 2 dpi with ~10^8^ DNA copies/µg of RNA. Virus production from CD24-low and -high cells correlated with the above differences in the appearance of Env+ cells over time. This is shown in Figure 1F, where titers of infectious ZIKV progeny from infected CD24-low cells remained at ~4–5 log PFU/mL across the time course, compared to the time-dependent increase in viral titer seen with infected CD24-high cells, which peaked at ~10^7^ PFU/mL by 3 dpi.

We tested the hypothesis that the low-level permissivity of CD24-low cells to ZIKV infection could be overcome by increased MOI. CD24-low and -high cells were infected with ZIKV at an MOI of 5, and Env+ and non-viable PI+ cells were quantified by flow cytometry. As shown in Figure 1G, an MOI of 5 did not change the expression of Env+ in CD24-low cells, with only ~5–10% of cells staining positive for Env expression. In contrast, an increase in Env+ expression was observed in ZIKV-infected CD24-high cells, with ~60% Env+ cells by 2 and 3 dpi (Figure 1G), and ~40% of CD24-high cells in the population showing PI+ stain by 3 dpi (Figure 1H). At an MOI of 100, less than 10% of the CD24-low cells stained positive for ZIKV Env+ expression, with minimal PI+ staining detected (data not shown). These data indicate that CD24-low cells are restricted for ZIKV infection, spread, and virus production (even at very high MOIs) and that these restrictions can be relieved by the ectopic expression of CD24.

### 3.2. STAT1 Is Constitutively Phosphorylated and Localized to the Nucleus in CD24-Low Cells

**Figure 2:**
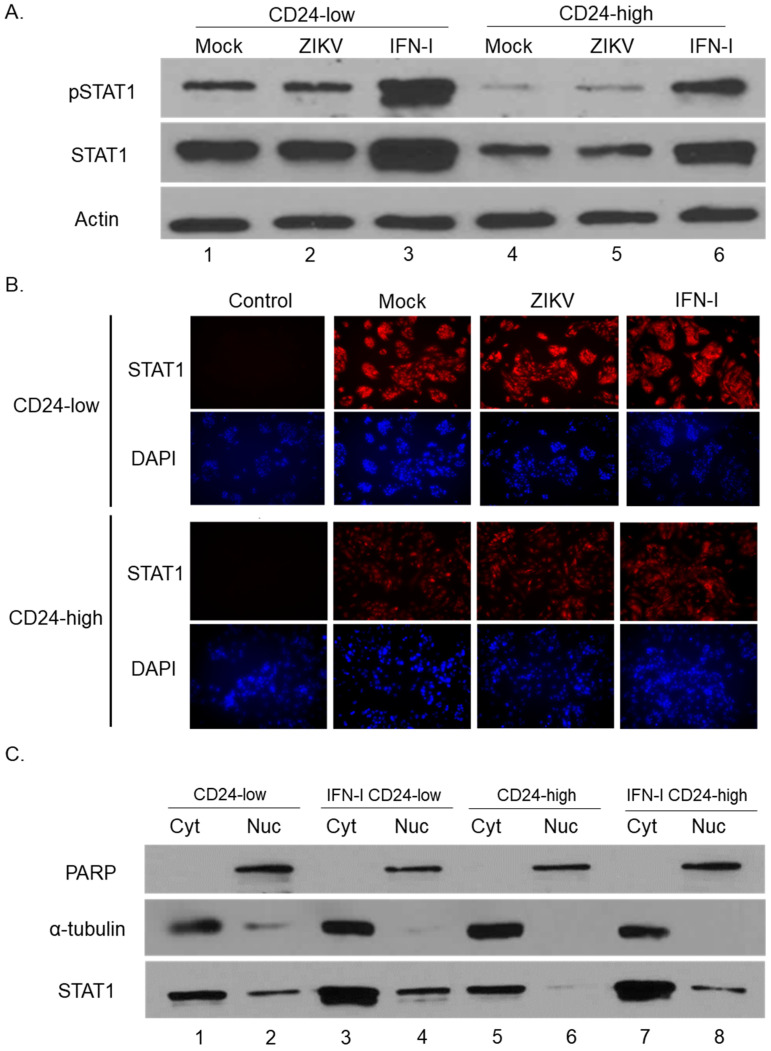
CD24-low and CD24-high cells differ in total levels, phosphorylation, and cellular distribution of STAT1. (**A**,**B**) Cultures of CD24-low and -high cells were either mock-infected, ZIKV-infected at MOI 5, or treated with 10 u/mL of IFN-I for 18 h. Levels of STAT1, pSTAT1, and actin proteins were analyzed by Western blotting (**A**). Alternatively, STAT1 levels were also determined by immunostaining (**B**) with an antibody to STAT1 (red) and nuclear DAPI (blue). Exposure times for immunofluorescence and DAPI were the same between mock- and ZIKV-infected CD24-low and CD24-high cells. Images are shown at 20× magnification. (**C**) CD24-low and -high cells were either left untreated or treated with 10 units/mL of IFN-I for 18 h. Cell lysates were fractionated into cytoplasmic (Cyt) and nuclear (Nuc) fractions before analysis by Western blotting for PARP as a nuclear marker, α-tubulin for a cytoplasmic marker, and total STAT1.

We hypothesized that the low permissivity of CD24-low cells to ZIKV infection was due to a strong, basal antiviral response. During some antiviral responses, STAT1 is activated through phosphorylation and subsequent translocation from the cytoplasm to the nucleus [2]. To determine levels of total and phosphorylated STAT1, CD24-low and -high cells were either mock-infected, infected with ZIKV at a high MOI, or treated with exogenous IFN-I (as a positive control) for 18 h. Protein levels were evaluated by Western blotting and by immunofluorescence. Consistent with our hypothesis, mock-infected CD24-low cells (Figure 2A, lane 1) had higher levels of total STAT1 and phosphorylated STAT1 (phosphorylated at Tyr701) than mock-infected CD24-high cells (lane 4). Densitometry analysis of the pSTAT1 band revealed mock-infected CD24-low cells (lane 1) had a high signal of 148,000, compared to a lower signal of 24,000 in mock-infected CD24-high cells (lane 4). Immunofluorescence confirmed this finding, where increased levels of STAT1 were observed in the mock-infected CD24-low cells compared to CD24-high cells (Figure 2B). The higher levels of STAT1 seen in mock-infected CD24-low versus -high cells are consistent with the basal phosphorylation of STAT1 in CD24-low cells, since STAT1 itself is an ISG [2].

Following ZIKV infection, CD24-low and -high cells showed no significant change in total STAT1 levels or STAT1 phosphorylation detected by Western blotting (Figure 2A, comparing lanes 1 to 2 and lanes 3 to 4) or by immunofluorescence (Figure 2B). IFN-I treatment increased STAT1 phosphorylation and overall levels, with slightly more activation in the CD24-low versus -high cells (Figure 2A, lanes 3 and 6).

We used cell fractionation to evaluate the effect of CD24 expression on basal distribution of STAT1 within the cell. CD24-low and -high cells were left untreated or treated with IFN-I (as a positive control), and cell lysates were separated into cytoplasmic (Cyt) and nuclear (Nuc) fractions, as described in Materials and Methods. Fractions were assayed by Western blotting for a nuclear control protein (PARP), a cytoplasmic control protein (α-tubulin), and total STAT1. As shown in Figure 2C, PARP was localized in nuclear fractions (lanes 2, 4, 6, and 8), while α-tubulin was localized mainly to cytoplasmic fractions (lanes 1, 3, 5, and 7). In untreated and IFN-I-treated CD24-low and -high cells, STAT1 was predominantly detected in the Cyt fractions (lanes 1, 3, 5, 7), with some levels of STAT1 found in the nuclear fraction after IFN-I treatment (lanes 4 and 8). Most importantly, in the absence of IFN-I, nuclear STAT1 was found in higher levels in CD24-low cells compared to CD24-high cells (compare lanes 2 and 6). Together, these data are consistent with the conclusion that CD24-low cells have basal constitutive phosphorylation of STAT1 and a detectable level of nuclear translocation, which is dampened by CD24 expression in the case of the CD24-high cells.

### 3.3. CD24 Expression Decreases the Transcriptional Response of CD24-Low Cells to IFN-I Stimulation and Increases Their Permissivity to RNA Virus Infection

**Figure 3:**
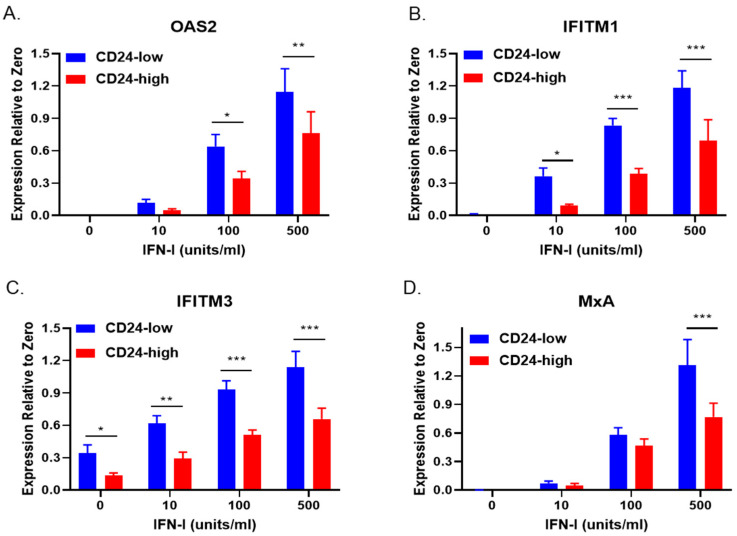
CD24-low and -high cells differ in ISG expression in response to IFN-I stimulation. CD24-low and -high cells were stimulated with the indicated concentrations of IFN-I for 16 h. Total cellular RNA was isolated, and levels of ISGs *OAS2* (**A**), *IFITM1* (**B**), *IFITM3* (**C**), and *MxA* (**D**) were determined by qRT-PCR. Data are expressed as basal gene levels (Expression Relative to Zero). Values are the mean of three samples, with error bars representing standard deviation. *, **, and *** indicate *p*-values < 0.033, <0.002, and <0.001, respectively.

Our above finding of constitutive STAT1 phosphorylation in CD24-low cells raised the hypothesis that CD24-low and -high cells would differ in their response to IFN-I stimulation. To test this, CD24-low and -high cells were treated for 16 h with increasing concentrations of exogenous IFN-I, as indicated in Figure 3. Total RNA was then isolated, and qRT-PCR was performed to determine increases in gene expression levels of *OAS2, IFITM1, IFITM3*, and *MxA*—four key ISGs known to play a role in establishing an antiviral state [2]. As shown in Figure 3A, there was a dose-dependent increase in IFN-I-stimulated expression of *OAS2,* which was significantly elevated in CD24-low compared to CD24-high cells. Similar results were seen for *IFITM1* (Figure 3B), *IFITM3* (Figure 3C), and *MxA* (Figure 3D), where IFN-I treatment resulted in higher basal gene expression in CD24-low cells compared to CD24-high cells. It is noteworthy that *IFITM1* and *IFITM3* expression have been shown to restrict ZIKV replication at an early step in replication [19].

**Figure 4:**
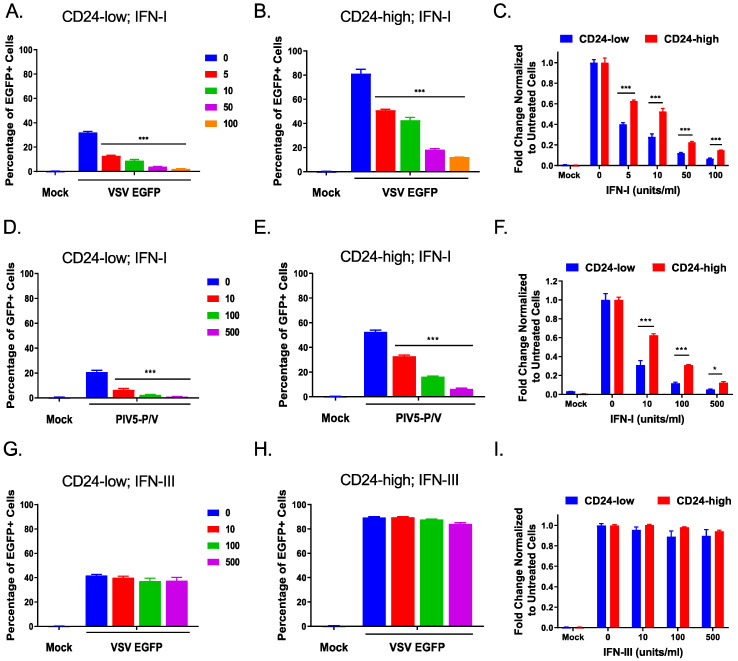
CD24-low and CD24-high cells differ in IFN-I-mediated sensitivity to virus infection. CD24-low (**A**,**D**,**G**) and CD24-high (**B**,**E**,**H**) cells were treated with the indicated concentrations of IFN-I (**A**–**F**) or IFN-III (**G**–**I**) for 14–17 h or 24 h, respectively. Treated cells were then either mock-infected or infected with VSV-EGFP at an MOI of 5 for 7 h (panels (**A**–**C**,**G**–**I**)), and flow cytometry was performed to determine the percentage of EGFP+ cells. Alternatively, IFN-I treated cells were infected at an MOI of 5 with the PIV5-P/V virus. Flow cytometry was performed ~24 h after infection (**D**–**F**) to determine the percentage of GFP+ cells. To compare the relative permissivity of IFN-treated CD24-low and -high cells to VSV-EGFP or PIV5-P/V, data are expressed as fold changes normalized to respective untreated cell populations (panels (**C**,**F**,**I**)). Values are the mean of three samples, with error bars representing standard deviation. * and *** indicate *p*-values of <0.033 and <0.001, respectively.

We used an infectivity assay to test the functional consequences of CD24-low cells having higher transcriptional responses to IFN-I stimulation compared to CD24-high cells. To achieve this, we assayed the functional antiviral state of CD24-low cells compared to CD24-high cells using vesicular stomatitis virus expressing EGFP (VSV-EGFP), a well-characterized prototypic RNA virus that is highly sensitive to IFN-I [20]. CD24-low and -high cells were exogenously treated with increasing concentrations of IFN-I and subsequently infected with VSV-EGFP at an MOI of 5. The percentage of infected EGFP+ cells was determined by flow cytometry at 7 h post-infection (hpi). As shown in Figure 4A, untreated CD24-low cells had only ~30% of the population as EGFP+, and treatment with 100 u/mL IFN-I resulted in <5% of CD24-low cells as EGFP+. This result contrasted with CD24-high cells, as shown in Figure 4B, where ~80% of untreated cells were EGFP+, and the decrease in EGFP+ cells following IFN-I treatment was less robust compared to CD24-low cells. To directly compare changes in IFN-I response between CD24-low and -high cells, the percentage of EGFP+ cells at each IFN-I dose was normalized to untreated infected cells within their respective populations. As shown in Figure 4C, CD24-low cells (blue bars) showed a significantly lower number of infected cells at all doses of IFN-I compared to CD24-high cells (red bars).

Similar results were observed when CD24-low and -high cells were pretreated with IFN-I and infected with a second IFN-I-sensitive virus—the Parainfluenza Virus 5 P/V mutant (PIV5-P/V) expressing GFP [10]. As shown in Figure 4D,E, untreated CD24-low and -high cells showed ~20% and 50% GFP+ cells, respectively, demonstrating an inherent difference in their permissivity to PIV5-P/V infection. Likewise, after IFN-I pre-treatment, there was a more robust restriction of PIV5-P/V infection in CD24-low compared to CD24-high cells (comparing Figure 4D to Figure 4E). This difference in IFN-I responsiveness with CD24-low cells was more evident when GFP+ cells were normalized to their respective untreated infected cell numbers (Figure 4F, blue bars) compared to CD24-high cells (Figure 4F, red bars).

To determine whether CD24-low and -high cells differed in their response to type III Interferon (IFN-III), cells were treated with exogenous IFN-III at increasing concentrations for 24 h and subsequently infected with VSV-EGFP. Flow cytometry analysis of EGFP+ CD24-low (Figure 4G) or CD24-high cells (Figure 4H) showed no significant responses in either cell line to exogenous IFN-III (Figure 4I). Taken together, these data indicate that CD24-low cells have a more robust transcriptional and functional response to IFN-I (but not IFN-III treatment) when compared to CD24-high cells.

### 3.4. IRF-1 and NFκB Levels Are Elevated in CD24-Low Cells Compared to CD24-High Cells

**Figure 5:**
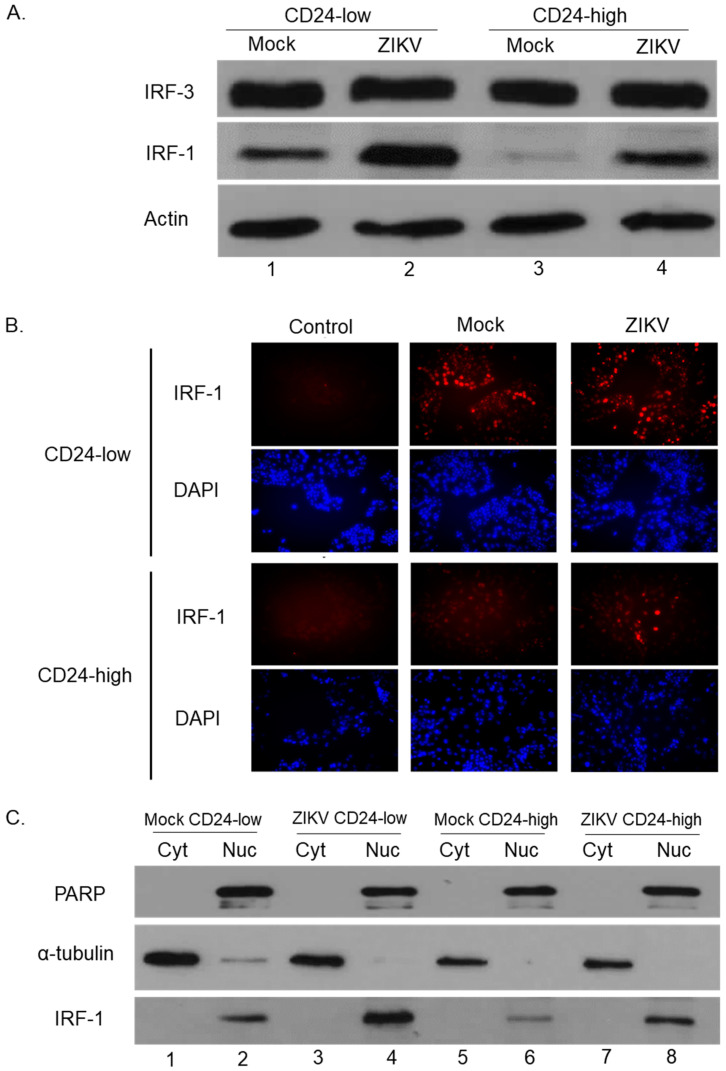
CD24-low and CD24-high cells differ in total levels of IRF-1 but not IRF-3. CD24-low and -high cells were either mock- or ZIKV-infected at MOI of 5 for 18 h. (**A**) Levels of total IRF-1, IRF-3, and actin proteins were analyzed by Western blotting. (**B**) Cultures of CD24-low and -high cells were either mock-infected or infected with ZIKV at MOI of 5 for 18 h. Levels of IRF-1 were determined via immunostaining with an antibody to IRF-1 (red) or DAPI nuclear staining (blue). Images are 20× magnification. (**C**) CD24-low and -high cells were either mock-infected or ZIKV-infected at MOI of 5 for 18 h. Cell lysates were fractionated into cytoplasmic (Cyt) and nuclear (Nuc) fractions before analysis by Western blotting for PARP as a nuclear marker, α-tubulin for a cytoplasmic marker, and total IRF-1.

IRF transcription factors (e.g., IRF-3, IRF-1) are essential mediators of antiviral pathways [2,21]. To determine whether total levels of IRF-1 and IRF-3 are affected by ectopic CD24 expression, mock- and ZIKV-infected CD24-low and -high cell lysates were analyzed by Western blotting. As shown in Figure 5A, levels of IRF-3 did not differ between CD24-low and -high cells, either at basal level (lane 1 and 3) or following ZIKV infection (lane 2 and 4). By contrast, mock-infected CD24-low cells had elevated levels of IRF-1 compared to CD24-high cells, which showed minimal to undetectable levels of IRF-1 (Figure 5A, comparing IRF-1 lanes 1 and 3). Densitometry analysis of the IRF-1 band in Figure 5A revealed that mock-infected CD24-low cells (lane 1) had a signal of 306,000, which was lower than the 128,000 in mock-infected CD24-high cells (lane 3). In both cell lines, IRF-1 was upregulated following ZIKV infection (Figure 5A, lanes 2 and 4), to signals of 548,000 and 394,000 in CD24-low and –high cells, respectively. These results were confirmed by immunostaining mock- and ZIKV-infected CD24-low and -high cells with anti-IRF1 Ab. As shown in Figure 5B, mock- and ZIKV-infected CD24-low cells showed robust IRF-1 staining, contrasting with CD24-high cells, which showed low levels of IRF-1 staining.

When activated, IRF-1 translocates into the nucleus to facilitate antiviral responses [22,23]. To determine whether CD24 expression affects cellular localization of IRF-1, cell lysates of mock- and ZIKV-infected CD24-low and -high cells were fractionated into their respective Cyt and Nuc fractions and analyzed by Western blotting for PARP (Nuc control), α-tubulin (Cyt control), and IRF-1. As shown in Figure 5C, PARP protein localized to the Nuc fractions (lanes 2, 4, 6, and 8), while α-tubulin localized mainly to the Cyt fractions (lanes 1, 3, 5, and 7). Basal and ZIKV infection-induced levels of IRF-1 were similarly fractionated in the case of CD24-low and -high cells, with the only apparent difference being the overall higher level of IRF-1 expression in CD24-low cells (Figure 5C, compare lanes 2 and 6).

**Figure 6:**
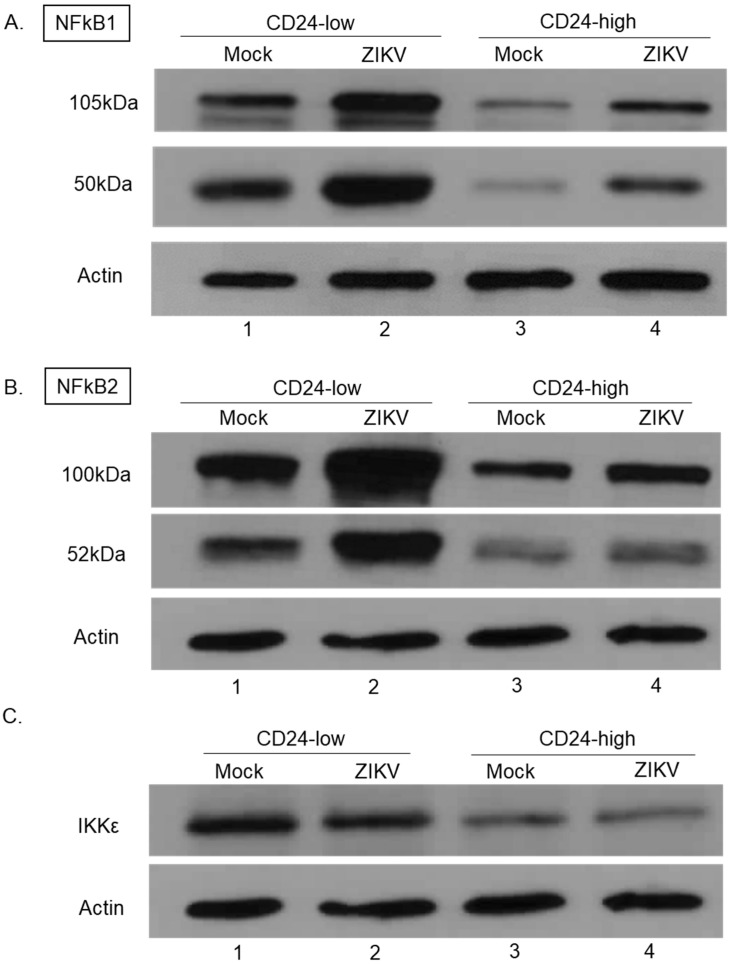
The expression of NFκB1, NFκB2, and IKKε differs between CD24-low and CD24-high cells. Cells were either mock- or ZIKV-infected at MOI of 5 for 18 h. Total levels of (**A**) NFκB1 (p105/p50), (**B**) NFκB2 (p100/p52), (**C**) IKKε, and actin were analyzed via Western blotting.

NFκB transcription factors can play an important role in induction of antiviral responses, including IFN-I [24]. NFκB1 (p105/p50) and NFκB2 (p100/p52) exist as precursors of p105 and p100, which are proteolytically processed into p50 and p52, respectively [24]. To determine whether levels of NFκB1 and NFκB2 differed between CD24-low and -high cells, Western blotting was performed on mock- and ZIKV-infected CD24-low and -high cells. As shown in Figure 6A, CD24-low cells had levels of precursor NFκB1 (p105) and processed protein p50 that were higher than those seen for CD24-high cells, both under basal and ZIKV-infected conditions (compare lanes 1 and 2 versus 3 and 4). Similarly, precursor levels of NFκB2 p100 and cleavage product p52 were elevated in CD24-low cells compared to CD24-high cells (Figure 6B).

We next evaluated the effect CD24 expression has on total levels of IKKε, the kinase commonly known to aid in phosphorylation of IRF-3 and IRF-1 [25,26]. As seen in Figure 6C, upregulated levels of IKKε were found in mock- and ZIKV-infected CD24-low cells compared to CD24-high cells (compare lanes 1 and 2 versus 3 and 4). These data indicate that ectopic CD24 expression results in reduced levels of both basal and ZIKV-induced NFκB1, NFκB2, and IKKε.

### 3.5. CD24-Low and -High Cells Do Not Differ in Basal IFN-I Production or Soluble Antiviral Mediators

**Figure 7:**
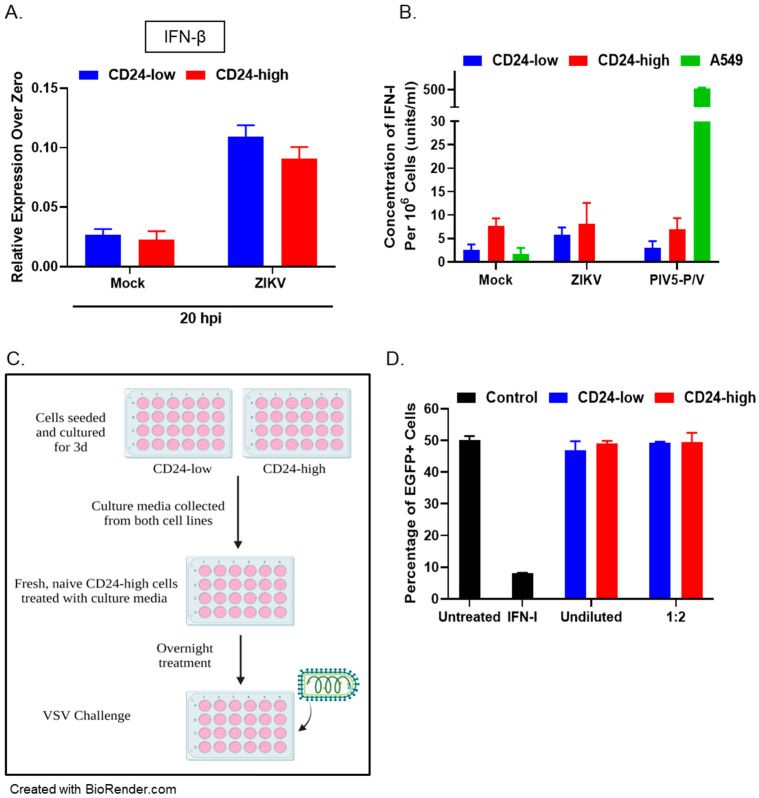
Soluble factors do not differentiate between the sensitivity of CD24-low and CD24-high cells to virus infection. (**A**) Cultures of CD24-low and -high cells were either mock-infected or ZIKV-infected at MOI of 5, and total cellular RNA was isolated at 20 hpi. Levels of *IFN-β* were evaluated at 20 hpi and are expressed as a relative expression over time zero (basal levels). (**B**) CD24-low and -high cells were either mock-infected or infected with ZIKV for 18 h. A549 cells that were mock-infected or infected with PIV5-P/V mutant at MOI of 5 were used as negative and positive controls for IFN-I production, respectively (green bars). The levels of IFN-I in culture media were quantified using a biological assay with HEK-Blue™ IFN-α/β cells as an indicator cell line (as described in Materials and Methods). Data are a representation of two separate experiments. (**C**,**D**) Media were collected from uninfected CD24-low and -high cells and either left undiluted or diluted 1:2 to treat naïve CD24-high cells for 16 h. As negative and positive controls, CD24-high cells were left untreated or treated with 100 u/mL IFN-I, respectively (black bars). Treated CD24-high cells were then infected with VSV-EGFP at MOI of 5, and at ~6 hpi, the percentage of EGFP+ cells was determined by flow cytometry. Values are the mean of three samples.

To evaluate the effect of CD24 expression on IFN-I induction, CD24-low and -high cells were either mock-infected or ZIKV-infected at an MOI of 5. At 20 hpi, RNA was extracted, and qRT-PCR was performed with primers specific for *IFN-β*. As shown in Figure 7A, there was no significant difference in basal levels (relative expression over zero) of *IFN-β* gene expression in mock-infected CD24-low and -high cells at 20 hpi.

A functional bioassay was used to determine whether active IFN-I was produced from CD24-low and -high cells. CD24-low and -high cells were either mock-infected or infected with ZIKV or the positive control PIV5-P/V virus, a known inducer of high levels of IFN-I [10]. Supernatant was collected at 18 hpi and subsequently treated to inactivate any virus present. IFN-I levels were then determined with indicator HEK-Blue™ IFN-α/β cells, which are used to quantify biological levels of IFN-I (as described in Materials and Methods). As shown in Figure 7B, minimal levels of IFN-I were detected in mock- and virus-infected CD24-low cells (blue bars), which was similar to corresponding conditions in CD24-high cells (red bars) and negative control mock-infected A549 cells (green bar). In contrast, the positive control PIV5-P/V A549 cell infection (green bar) yielded ~500 u/mL of IFN-I.

A media-transfer experiment was conducted to test the hypothesis that soluble antiviral mediators produced basally by CD24-low cells (including low levels of IFN-I) could have antiviral properties. As shown schematically in Figure 7C, culture media were taken from uninfected CD24-low and -high cells and either left undiluted or diluted at a 1:2 ratio. Fresh, naïve CD24-high cells were then treated with these culture samples for ~16 h before infection with VSV-EGFP at MOI 5. Virus-infected EGFP+ cells were detected by flow cytometry at ~6 hpi. As controls, CD24-high cells were either left untreated (Figure 7D, black bars, negative control) or were treated with exogenous IFN-I (black bar, positive control) before their infection with VSV-EGFP. As shown in Figure 7D, untreated CD24-high cells showed ~50% EGFP+ cells, which was reduced by IFN-I treatment to ~10% infected EGFP+ cells. After treatment with culture medium from either CD24-low or -high cells, ~50% of CD24-high cells were EGFP+ cells, similar to the untreated control. These results support the proposal that differences in permissivity of CD24-low or -high cells to virus infection are not due to differences in the baseline production of soluble antiviral mediators.

### 3.6. Ectopic Expression of CD24 Alters the Global Transcriptomic Profile of Intracellular Signaling Pathways

**Figure 8:**
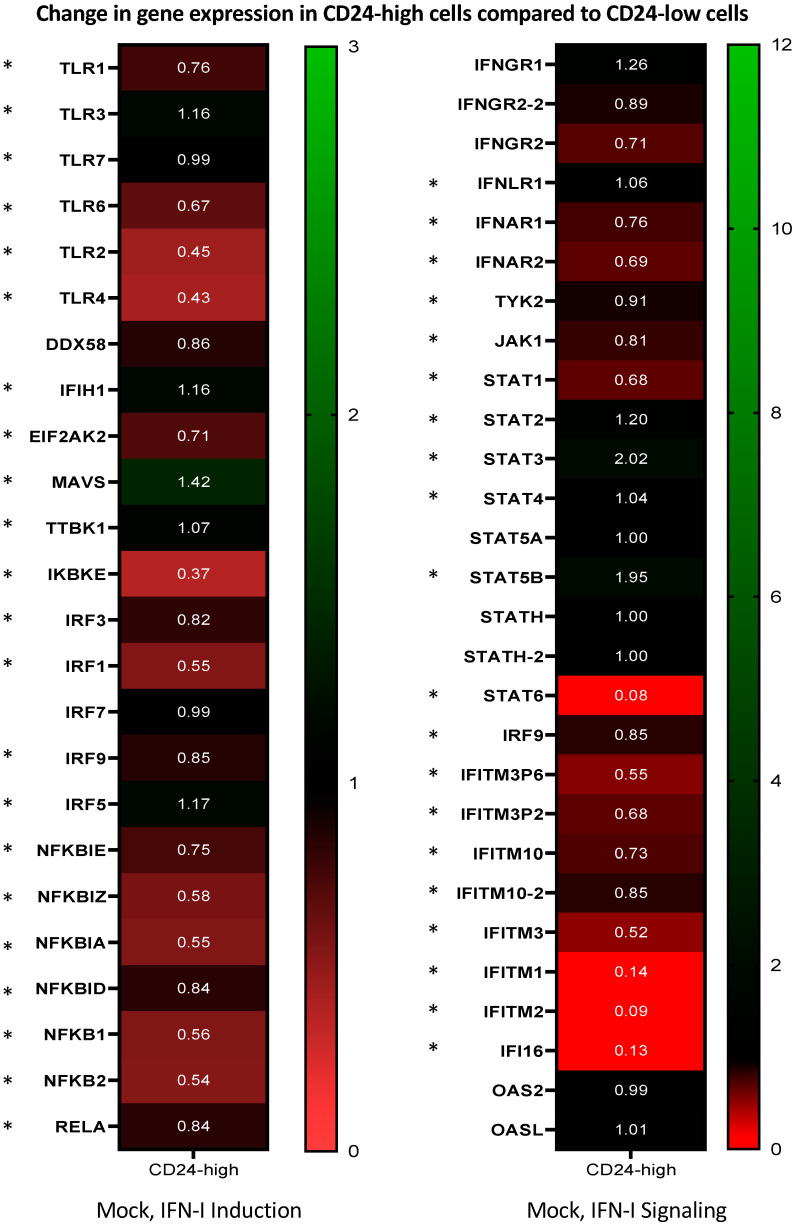
Global transcriptomic analysis reveals downregulation of key IFN-I pathway genes in CD24-high versus CD24-low cells. In the absence of virus, cultures of CD24-low and -high cells were mock-infected, and total cellular RNA was isolated and analyzed by RNA sequencing as described in the Materials and Methods. Using CD24-low cells as a baseline, heat maps were generated to visualize fold changes in IFN induction and IFN-signaling pathway genes that were either upregulated (green, values > 1) or downregulated (red, values < 1) in mock-infected CD24-high cells. Genes labelled with * indicate a *p*-value less than 0.05. Values are the mean of three samples.

The above data support the proposal that CD24-low cells have an elevated basal antiviral state and that ectopic expression of CD24 dampens these responses. To determine the overall antiviral state of CD24-low and -high cells in the absence of virus infection, total RNA was extracted from mock-infected CD24-low and -high cells (at 20 hpi), and global transcriptomics were profiled via RNA sequencing as described in the Materials and Methods. Transcriptional levels of vital mediators and effectors of both the IFN-I induction and signaling pathways were analyzed in mock-infected CD24-low and -high cells and displayed as a heat map of fold change differences for the indicated genes. As shown in Figure 8, an overall trend of reduced gene expression was observed in IFN-I induction mediators (*TLR* family, *NFκB* family, *IRF* family) in the transcriptomic profiles of CD24-high cells compared to CD24-low cells. Similar trends were observed in levels of critical IFN-I signaling genes (*IFNAR*, *JAK1*, *TYK2*, *STAT1*) and ISGs (*IFITM* family) [2,27,28]. These data suggest that key induction and signaling mediators of the IFN-I pathway are transcriptionally downregulated by ectopic CD24 expression in mock-infected CD24-high cells, thereby indicating a global role of CD24 in repressing basal host innate antiviral responses.

We extended our studies to observe transcriptional changes of key IFN-I induction and signaling mediators during ZIKV infection (at 20 and 40 hpi). Additional heat maps were generated to analyze the fold change of genes indicated in Figure 8 as part of a longitudinal study, which included ZIKV-infected CD24-low and -high cells (at 20 and 40 hpi) (Figures S1 and S2). Figure S1 shows trends of upregulated levels of genes in the IFN-I induction pathway in ZIKV-infected CD24-low and -high cells at 40 hpi. Certain genes appeared to have higher expression levels in infected CD24-high cells (e.g., *IRF-1*, *IRF-7*, and a few members of the *NFκB* family) compared to CD24-low cells. This is likely due to the higher permissivity of CD24-high cells to ZIKV infection. ZIKV-infected CD24-low cells showed a greater number of significant and modulated genes in the IFN-I signaling phase, compared to CD24-high cells (Figure S2). However, gene expression levels of the *IFITM* family in ZIKV-infected CD24-high cells at 40 hpi are robustly upregulated, once again likely due to the enhanced ZIKV infection in CD24-high cells.

To determine how CD24 expression affects host intracellular signaling pathways, we performed a reactomeFI pathway network enrichment that included significant changes in genes observed upon ectopic CD24 expression (in CD24-high cells). As shown in Figure S3, the pathways that have been significantly modulated belong to various intracellular signaling pathways, including IFN-α/ß signaling, JAK-STAT signaling, and Ras signaling, and also host cellular networks, such as extracellular matrix (ECM) organization. Most genes involved in Ras signaling were significantly upregulated, while the majority of genes involved in ECM organization appeared to be downregulated (Figure S4). Upon ectopic CD24 expression, the gene *CD44* (encoding cell surface adhesion receptor CD44) was downregulated, which has been shown to be expressed in cancer cells and regulates tumor cell invasion and metastasis [29] (Figure S4). Additionally, integrin subunit α genes (*ITGA1* and *ITGA8*) appeared to be significantly downregulated, along with Collagen, type IV, α genes (*COL4A1*, *COL4A2*, *COL4A6*). Certain collagen genes were highly upregulated, for example Collagen, type V, α (*COL5A2*) was upregulated ~34 fold upon CD24 expression. Figure S4 also shows an upregulation of genes involved in the activation of Ras signaling: a neurotrophic tyrosine kinase receptor (NTRK1, upregulated ~2.75 fold), Fibroblast growth factor 10 (FGF10, upregulated ~4.68 fold), and Insulin-like growth factor 2 (IGF2, upregulated ~2.8 fold) [30,31,32], indicating a dysregulated Ras signaling transcriptome upon ectopic CD24 expression.

Taken together, the transcriptomics data indicate that CD24 expression has a global effect on basal and virus-induced levels of gene transcripts involved in various intracellular signaling pathways associated with antiviral responses.

## 4. Discussion

Our prior work showed that ZIKV could infect a range of human neuroblastoma cell lines, with the exception of SK-N-AS cells, which were highly restricted for infection [7]. Notably, while our published work showed that this ZIKV restriction in SK-N-AS cells could be relieved by ectopic expression of cellular CD24, the mechanisms for differences in ZIKV replication in the parental SK-N-AS and CD24-expressing cells were unclear. Given that IFN-I has been shown to play a role in influencing ZIKV infectivity [33,34,35,36,37], and it can be a major determinant of tissue specificity for many virus infections [2], we tested the hypothesis that CD24-low and -high cells differed in antiviral responses. Here, we showed that SK-N-AS cells with poor CD24 expression have a heightened basal level of an antiviral state, which is evident by their: (i) increased sensitivity to exogenous IFN-I, (ii) constitutive levels of phosphorylated STAT1, and (iii) high-level expression of antiviral genes, such as IRFs and NFκB. Most strikingly, our data show that ectopic expression of CD24 increases the permissivity of CD24-high cells to infection with three different RNA viruses—ZIKV, VSV, and PIV5-P/V. Together, these data support our model that CD24 expression dampens the overall basal antiviral state of neuroblastoma cells through changes to intracellular factors that affect permissivity to virus infection.

While the physiological cellular receptor for ZIKV has not been identified, prior studies have suggested the tyrosine kinase receptor AXL can influence ZIKV entry [6]. Our prior work showed that differential infectivity across a range of neuroblastoma cell lines to ZIKV infection did not correlate with AXL expression [7], and our transcriptional data here indicate that CD24-low and -high cells do not differ in the levels of AXL expression. Strikingly, we found that CD24-low cells show low permissivity profiles for ZIKV but also for VSV-EGFP and the PIV5-P/V mutant [10], and permissivity to each of these viruses was enhanced by ectopic CD24 expression. Since VSV utilizes the LDL receptor and PIV5 binds to cell surface sialic acid as a receptor [38,39], it is unlikely that CD24 itself acts as a receptor per se for increasing virus entry between CD24-low and CD24-high cells.

The permissivity of CD24-low cells to infection with ZIKV, PIV5-P/V, and VSV-EGFP differed substantially. ZIKV was highly restricted, with only ~10% cells showcasing infection when exposed to a high MOI (Figure 1). PIV5-P/V exhibited higher permissivity, with ~20% of cells infected when exposed to an MOI of 5. VSV was the least restricted of these three viruses, with ~30–40% of cells infected at a high MOI (Figure 4). Permissivity for all three viruses was increased in the CD24-high cells: ZIKV and PIV5-P/V to ~50–60% and VSV-EGFP to ~80% of cells being infected (Figure 1 and Figure 4). Why does CD24 expression have differing effects on permissivity to infections with these three RNA viruses? Prior work has shown that distinct subsets of activated ISGs are impactful for determining the outcome of a particular virus infection: IFITM1 and IFITM3 restrict ZIKV at an early step in replication [19]; IFITM3, Tetherin, and TRIM69 have been shown to inhibit VSV infection and replication [40,41]; and PKR and IFIT1 can restrict translation of PIV5 mRNAs [42,43]. Conversely, viruses counteract the antiviral state through different antagonists: the M protein of VSV [44], the V protein of PIV5 [45], and the NS5 protein of ZIKV [46]. Thus, this range of permissivity for different viruses in CD24-low cells and differences in the ability of CD24 expression to relieve restricted replication likely reflects the combination of the cellular landscape of antiviral genes being expressed and the activity of viral antagonists.

Phosphorylation of STAT1 is a critical step in the activation of ISG expression [2]. Strikingly, we showed here that CD24-low cells have both a constitutive baseline level of phosphorylated STAT1 and increased levels of STAT1 in the nucleus—both of which are dampened by ectopic CD24 expression. In breast cancer cells, CD24 expression has been shown to downregulate total and phosphorylated levels of STAT1 [47]. Consistent with this basal STAT1 activation, CD24-low cells were more sensitive to IFN-I-induced ISG expression and to entering an antiviral state with exogenously added IFN-I than was seen with CD24-high cells. While IFN-I and IFN-III share some of the same signaling components [48], we were not able to determine the effect of CD24 expression on IFN-III signaling, since SK-N-AS cells do not respond to exogenously added IFN-III.

How could CD24 alter cells to reduce their responsiveness to exogenous IFN-I? Prior studies have shown that CD24 is capable of changing signaling pathways at the plasma membrane through recruitment of specific proteins, such as β1 integrin, into lipid rafts that are rich in cholesterol and sphingolipids [49] or by selective exclusion of the CXCR4 chemokine receptor from plasma membrane lipid rafts [50]. In other examples, CD24 can regulate associations with danger-associated molecular pattern (DAMP) proteins, such as HMGB1 and immune cell receptor Siglec-G, resulting in altered Toll-like Receptor activity [51,52]. These findings indicate that CD24 is capable of modifying downstream signaling processes by altering the organization of the plasma membrane, as well as interacting directly with certain proteins [53]. Our RNA transcriptomics data suggest that genes involved in ECM organization are affected by ectopic CD24 expression (Figure S4), further highlighting a potential effect of CD24 expression on the plasma membrane network. Modifications to the plasma membrane, either in composition or curvature, have also been shown to impact viral entry [54,55]. Given that key mediators of IFN-I signaling are located in the plasma membrane (e.g., IFNAR1, Tyk2), it is possible that CD24 expression alters the composition or structure of key sites within the plasma membrane, which indirectly alters steps in IFN-I responses to virus infections [2]. Future studies will focus on determining the mechanism by which CD24 is capable of dampening IFN-I signaling responses.

While IFN-I signaling induced by exogenously added IFN-I differed between CD24-low and -high cells, our media-transfer experiments (Figure 7D) and direct measurements of IFN-I (Figure 7B) showed that the antiviral state of CD24-low cells was not dependent on a secreted factor. Thus, there was no detectable difference in the basal levels of IFN-I produced from CD24-low versus CD24-high cells, and culture medium from either cell line in the absence of virus was not able to induce an antiviral response in naïve cells. These data suggest that, in the absence of virus, the differential basal antiviral states in CD24-low versus CD24-high cells are not due to a secreted factor, but rather may be due to changes in intracellular factors. Consistent with this finding, we observed a robust expression of total IRF-1 in CD24-low cells, which was downregulated with increased CD24 expression (Figure 5A). Constitutive expression of IRF-1 in respiratory epithelial cells has been previously shown to establish an antiviral state in an IFN-I-independent manner by driving the expression of a distinct panel of antiviral genes (e.g., *OAS2, BST2*, and *RNASEL*) [3].

Our data indicate that CD24 expression also reduced levels of the non-canonical IκB kinase-ε (or IKKε), as well as NFκB1 and NFκB2 (Figure 6). Studies have shown that IKKε can play a role in IFN-I signaling, specifically by modulating phosphorylation of STAT1 [56,57]. NFκB is involved in multiple intracellular pathways that affect proliferation, apoptosis, and antiviral responses [24]. Prior work indicates that CD24 expression can decrease NFκB-driven signaling activities [58]. In the classical IFN-I pathway, NF-κB plays a key role in IFN-I induction, ultimately resulting in the expression and secretion of IFN-I from the cell. However, it can also directly induce expression of certain ISG subsets (*ISG15*, *IFIT1*) while repressing others (*Mx1*, *NMI*) [59]. Our future studies will focus on the complex relationship between NF-κB, IKKε, and IFN-I signaling in the context of CD24 expression.

Our results have implications for the possible use of CD24 as a biomarker for permissivity to viral infections, at least in neuroblastoma cells [7]. Further investigation will reveal whether these findings can be extended to other cells and tissues that show natural infection with ZIKV [6]. CD24 protein is expressed in certain cells of the skin (fibroblasts, keratinocytes, epidermal cells), brain (neuronal and glial cells), testis (Leydig cells and cells in seminiferous ducts), and in prostate and seminal vesicles (glandular cells) at moderate to high levels. In the female reproductive system, while the cells in the placenta express low levels of CD24, cells from the endometrium and fallopian tube express moderate to high levels of CD24 as well [60].

While being present in varying levels in different healthy cells and tissues, CD24 is also found to be upregulated in some cancer cells, with studies suggesting it to be an important marker for prognosis and diagnosis for certain types of tumor cells [8]. Since the approval of IMLYGIC™ (talimogene laherparepvec) by the Food and Drug Administration, there is a growing interest in utilizing oncolytic viruses (OVs) as viable tumor therapy [61,62]. The potential use of ZIKV as an OV therapy has been shown for glioblastoma tumors [63,64,65], but the direct analysis of the role of CD24 expression in OV therapy has not been established. Studies with OVs such as reovirus and modified herpes simplex virus-1 have shown a preference for replication and oncolysis in cancer cells with activated Ras signaling pathways [66,67,68,69]. Since our transcriptomics data here indicate that CD24 expression alters Ras signaling in SK-N-AS cells (Figure S4), there is a need to further study how CD24 expression influences the oncolysis and mechanism of action of OVs currently in clinical trials (Newcastle Disease Virus, reovirus, measles virus, HSV-1, etc.) [70].

In conclusion, our results show that the expression of CD24 in a neuroblastoma cell line alters both the basal antiviral state of the cells and responses to IFN-I, resulting in increased permissivity to infection by three different RNA viruses. These findings have important implications for understanding cellular factors in virus replication as well as the design of new and more selective oncolytic viral vectors.

## Figures and Tables

**Table 1:** Primers used in PCR.

Gene	Forward Primer	Reverse Primer
*GAPDH*	5′-TTAAAAGCAGCCCTGGTGAC-3′	5′-CTCTGCTCCTGTTCGAC-3′
*Ns1*	5′-GCCATCACAATACCAGAGAG-3′	5′-GGCCTTATCTCCATTCCATAC-3′
*IFN-β*	5′-CAGCTCTTTCCATGAGCTACAA-3′	5′-CAGTATTCAAGCCTCCCATTCA-3′
*IFITM3*	5′-ACCATTCTGCTCATCGTCATC-3′	5′-GAAGTTGGAGTACGTGGGATAC-3′
*OAS2*	5′-AGAAGCTGGGTTGGTTTATC-3′	5′-GACGTCACAGATGGTGTTC-3′

## Data Availability

Not applicable.

## Supplementary Materials

The following supporting information can be downloaded at: https://www.mdpi.com/article/10.3390/v14081735/s1. Figure S1: IFN-I induction genes are altered after ZIKV infection of CD24-low and -high cells; Figure S2: IFN-I signaling genes are altered after ZIKV infection of CD24-low and -high cells; Figure S3: CD24 expression results in modulation of diverse intracellular signaling pathways; Figure S4: CD24 expression modulates Ras signaling and extracellular matrix organization.
