# Supplementary material for: CD24 Expression Dampens the Basal Antiviral State in Human Neuroblastoma Cells and Enhances Permissivity to Zika Virus Infection
Source: Viruses. 2022 Aug 6;14(8):1735. doi: 10.3390/v14081735 (PMC9416398; doi:10.3390/v14081735)
Supplement: Supplementary file 1 [file viruses-14-01735-s001.zip › viruses-1837881-supplementary.pdf]

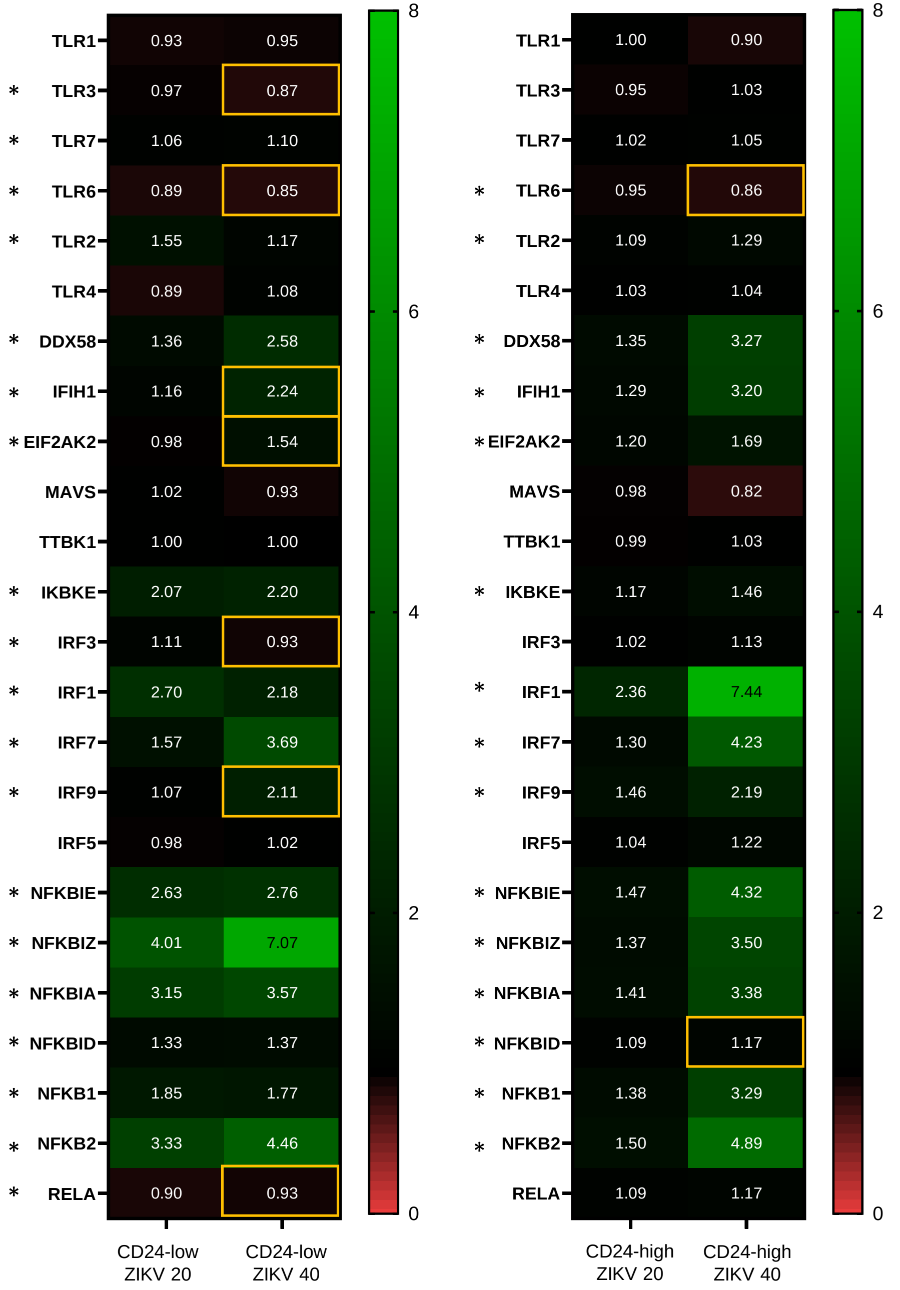

Figure S1. IFN-I induction genes are altered after ZIKV infection of CD24-low and -high cells. Total RNA from cultures of ZIKV infected CD24-low and -high cells at 20 and 40 hpi was isolated to perform RNA Sequencing as described in the Materials and Methods. Heatmaps were generated to visualize fold change of IFN-induction phase genes that were either upregulated (green, values >1) or downregulated (red, values <1) in ZIKV infected CD24-low and -high cells at 20 and 40 hpi. Genes labelled with "*" indicated statistical significance with a p-value less than 0.05. Statistically significant genes only at one time point are outlined in yellow. Values are the mean of three samples.

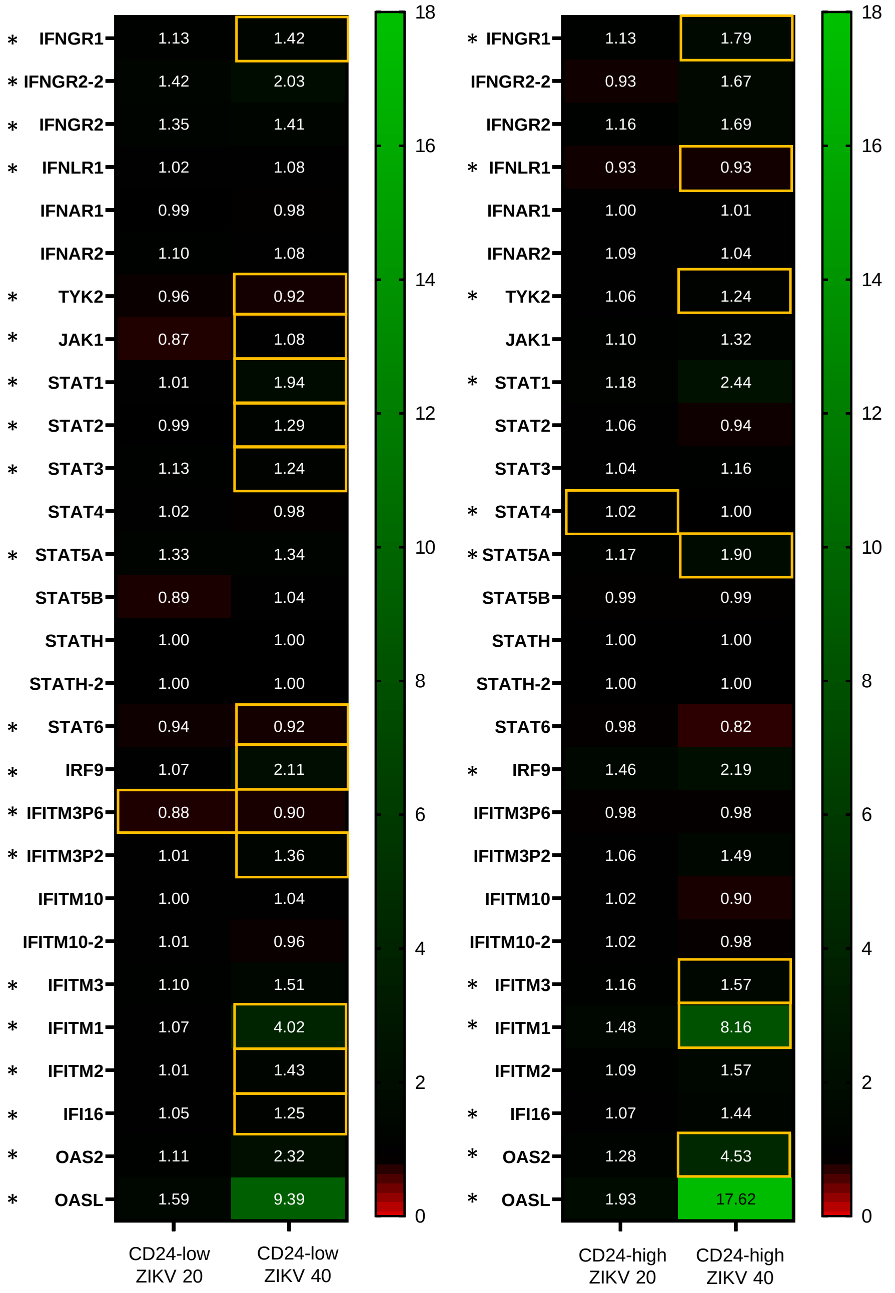

Figure S2. IFN-I signaling genes are altered after ZIKV infection of CD24-low and -high cells. Total RNA from cultures of CD24-low and -high cells infected with ZIKV was isolated at 20 and 40 hpi to perform RNA Sequencing (as described in the Materials and Methods). Heatmaps were generated to visualize fold change of IFN-signaling phase genes that were either upregulated (green, values >1) or downregulated (red, values <1) in of ZIKV infected CD24-low and -high cells at 20 and 40 hpi. Genes labelled with "*" indicated statistical significance with a p-value less than 0.05. Statistically significant genes only at one time point are outlined in yellow. Values are the mean of three samples.

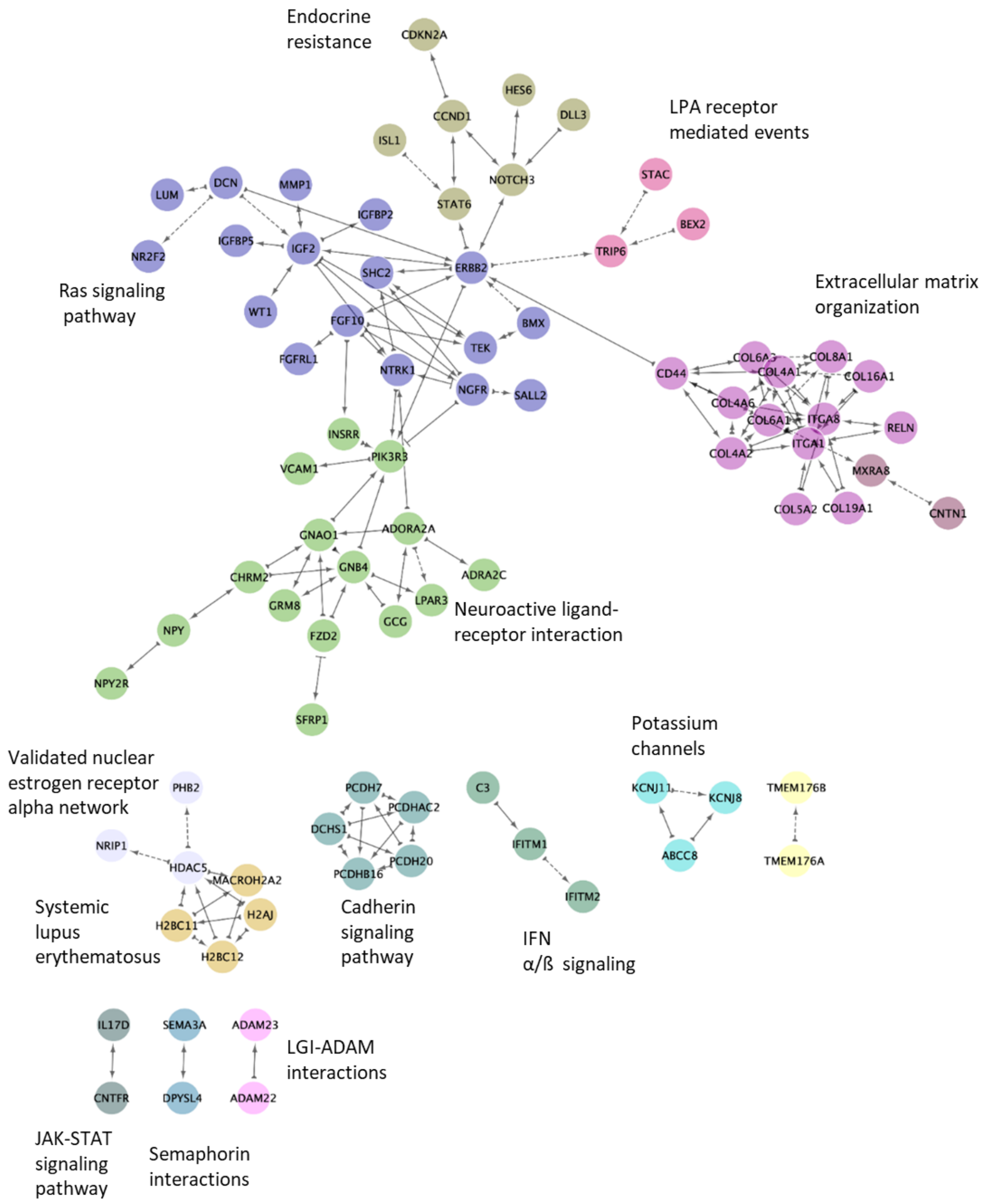

Figure S3. CD24 expression results in modulation of diverse intracellular signaling pathways. Pathway enrichment analysis of genes that were significantly modulated (as per conditions outlined in the Materials and Methods) was analyzed and plotted as different pathway modules, with opaque arrows indicating well established interactions and broken arrows indicating predicted interactions.

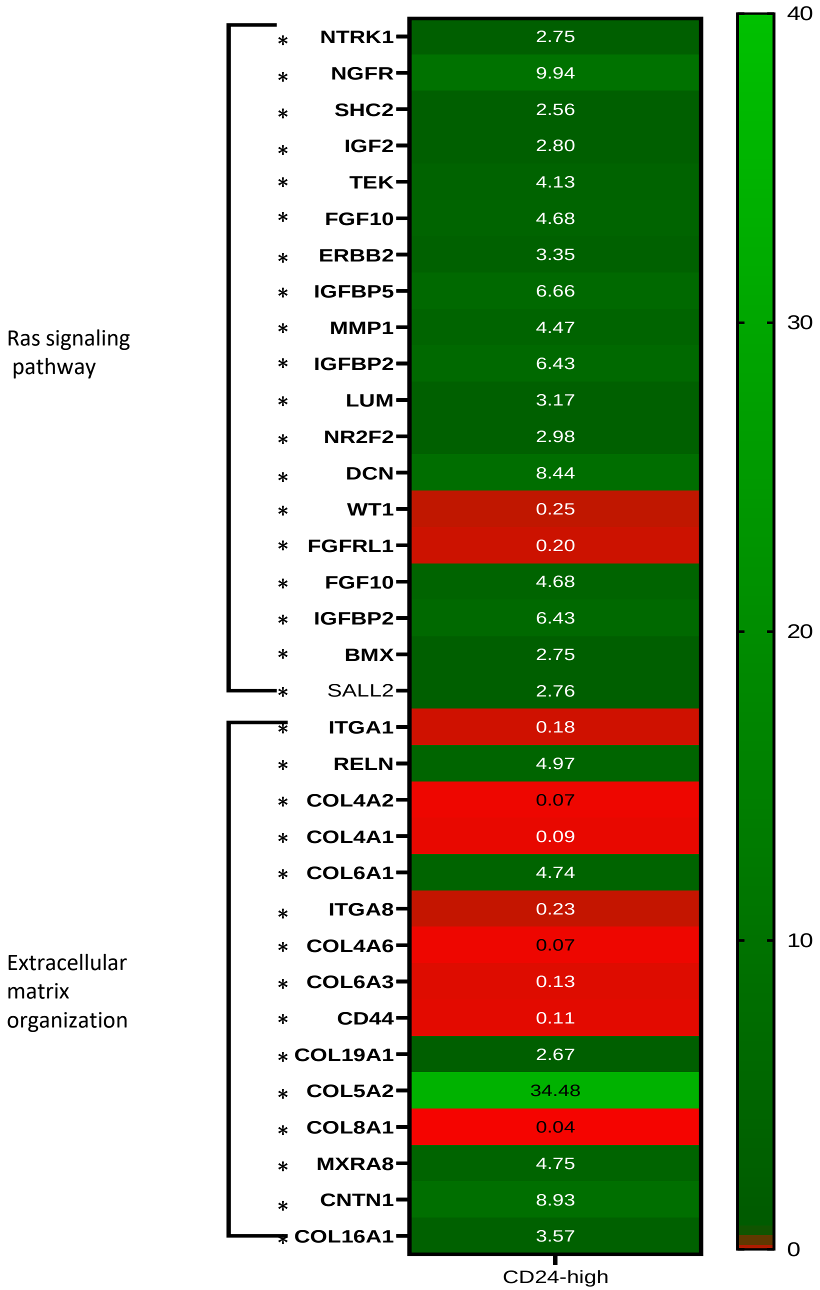

Figure S4. CD24 expression modulates Ras signaling and extracellular matrix organization. Total RNA from mock infected cultures of CD24-low and -high cells was isolated to perform RNA sequencing as described in the Materials and Methods. Heatmaps were generated to visualize fold change of genes that were either upregulated (green, values >1) or downregulated (red, values <1) in Ras signaling pathway and extracellular matrix organization in mock infected CD24-high cells compared to CD24-low cells. Genes labelled with "*" indicated statistical significance with a p-value less than 0.05. Values are the mean of three samples.
